# The Impact of Gender Norms on Condom Use among HIV-Positive Adults in KwaZulu-Natal, South Africa

**DOI:** 10.1371/journal.pone.0122671

**Published:** 2015-04-08

**Authors:** Kristin Fladseth, Mitzy Gafos, Marie Louise Newell, Nuala McGrath

**Affiliations:** 1 London School of Hygiene and Tropical Medicine, London, United Kingdom; 2 Medical Research Council Clinical Trials Unit, University College London, London, United Kingdom; 3 Africa Centre for Health and Population Studies, University of KwaZulu-Natal, Mtubatuba, South Africa; 4 Faculty of Medicine and of Social and Human Sciences, University of Southampton, Southampton, United Kingdom; University Hospital Basel, SWITZERLAND

## Abstract

Critical to preventing the spread of HIV is promoting condom use among HIV-positive individuals. Previous studies suggest that gender norms (social and cultural constructions of the ways that women and men are expected to behave) may be an important determinant of condom use. However, the relationship has not been evaluated among HIV-positive women and men in South Africa. We examined gender norms and condom use at last sex among 550 partnerships reported by 530 sexually-active HIV-positive women (372) and men (158) who had sought care, but not yet initiated antiretroviral therapy in a high HIV-prevalence rural setting in KwaZulu-Natal, South Africa between January 2009 and March 2011. Participants enrolled in the cohort study completed a baseline questionnaire that detailed their socio-demographic characteristics, socio-economic circumstances, religion, HIV testing history and disclosure of HIV status, stigma, social capital, gender norms and self-efficacy. Gender norms did not statistically differ between women and men (p = 0.18). Overall, condoms were used at last sex in 58% of partnerships. Although participants disclosed their HIV status in 66% of the partnerships, 60% did not have knowledge of their partner’s HIV status. In multivariable logistic regression, run separately for each sex, women younger than 26 years with more equitable gender norms were significantly more likely to have used a condom at last sex than those of the same age group with inequitable gender norms (OR = 8.88, 95% CI 2.95–26.75); the association between condom use and gender norms among women aged 26+ years and men of all ages was not statistically significant. Strategies to address gender inequity should be integrated into positive prevention interventions, particularly for younger women, and supported by efforts at a societal level to decrease gender inequality.

## Introduction

In 2013, over 6 million adults were living with HIV in South Africa [1]; numbers are increasing following the scale-up of antiretroviral therapy (ART) [2]. In South Africa, KwaZulu-Natal has the highest adult HIV prevalence at an estimated 29% among adults aged 15–49 years old in a rural area in 2011 [2]. Early initiation of ART reduces the risk of onward HIV transmission [3]. Currently in South Africa, treatment is initiated at <350 cells/μl [4] but the CD4 threshold will be raised to <500 cells//μl in January 2015 [5]. While an estimated 75% of eligible individuals received ART in South Africa in 2011, overall less than a third of HIV-positive adults are on treatment [6]; as such, condom use remains an important positive prevention strategy.

Reported condom use among HIV-positive women and men in South Africa is higher than in the general population [7–12]. By 2005, in rural KwaZulu-Natal, HIV-positive women were already significantly more likely to report using a condom with a regular partner at last sex than HIV-negative women [13]. In South Africa, higher levels of condom use among HIV-positive women and men have been associated with being male, younger age, higher education, and urban residency [7,10,14]. Condom use has also been linked to HIV-related factors including knowledge of being HIV-positive, longer duration since diagnosis, initiating ART, disclosure of HIV status to a partner and knowing a partner is HIV-negative [8,10,13,15–20]. HIV-positive adults’ lack of condom use has been associated with having a casual partner, sex with a positive partner, alcohol use ever or before sex, substance abuse in the past month or before sex, a history of forced sex (for women and men), and with coping strategies characterised by HIV denial and HIV-related stigma [7,9,11,14,19].

Despite this evidence, few studies have evaluated the impact of gender norms on condom use among HIV-positive women and men in South Africa. Gender norms, interpreted as social and cultural constructions of the ways that women and men are expected to behave, have been identified as important social drivers of the HIV epidemic [21–25], with implications for HIV prevention strategies for both women and men. For women, gender norms can create and reinforce their unequal position in relationships, families, societies and public domains [26,27]. A recent South African literature review demonstrated that women’s relative disempowerment in relationships with men reduced their ability to refuse sexual advances and negotiate safer sexual practices including condom use [26]. Attempts to refuse sex or insist on safer sex can result in verbal, economic, psychological, physical, or sexual abuse [22,28,29]. For men, gender norms can exacerbate concepts of masculinity that promote sexual prowess, virility, and male control over women, and frame condom use and fidelity as unmasculine [30–32]. However, in the post-Apartheid era, there has been growing evidence of increasing expectations of gender equality that are reshaping the gender norms that inform HIV prevention behaviours [29,33].

We investigated the impact of gender norms on condom use at last sex among a cohort of HIV-positive, ART-naïve women and men seeking HIV care at three primary health care clinics in rural KwaZulu-Natal.

## Methods

### Study design

The Hlabisa HIV Treatment and Care Programme is a partnership between the Department of Health (in 17 Primary Health Care clinics, (PHCs)) and the Africa Centre for Health and Population Studies in rural KwaZulu-Natal, South Africa [34]. The programme began in late 2004 and by December 2011, 20,598 adults had initiated treatment, an estimated 31% of all HIV-infected adults aged 15–49 years [2]. The Africa Centre Demographic Surveillance Area (ACDSA) is contained completely within the catchment area of the programme (see www.africacentre.ac.za).

The analyses presented here use baseline data from 632 individuals enrolled between January 2009 and March 2011 in a cohort study investigating the impact of ART on family and partner relationships and sexual behaviour of HIV-positive individuals. Details of the study design and baseline characteristics of the cohort have been described elsewhere [35,36]. Men and non-pregnant women diagnosed as HIV-positive, accessing the HIV treatment and care programme in three of the 17 PHCs, aged 18 years or older and resident within the ACDSA, were screened for study eligibility when they returned to the clinic to receive their CD4 test result. Individuals with CD4<200 cells/μl or WHO Stage IV HIV disease, consistent with national guidelines for ART-eligibility in 2009, and those with CD4>500 cells/μl were eligible to enrol in the study. The CD4>500 cells/μl cut-off was chosen to identify a group of HIV-diagnosed individuals that could be expected to have repeated measures over time before becoming ART-eligible [35]. A general introduction to the study was given each morning by study staff in the clinic waiting room and interested individuals were invited to approach the staff member. All those who met the staff member and met the eligibility criteria were invited to participate and taken through the study information sheet and informed consent process. Condom use was not a requirement for ART eligibility. Analyses were conducted on all sexually-active participants, irrespective of ART-eligibility at baseline.

Detailed information was collected on socio-demographic characteristics, socio-economic circumstances, religion, HIV testing history and disclosure of HIV status, stigma, social capital, gender norms and self-efficacy. Information regarding each of the three most recent sexual partners in the past six months was also ascertained; if the participant had not had a sexual partner in the six previous months, questions were asked about their most recent sexual partner. Participants who reported an ongoing partnership at baseline were asked additional questions about their fertility intentions with their current main partner or partners and the quality of those relationships, including questions about communication, conflict, stability, identity, and commitment [35].

Gender norms were measured by a set of 19 questions (appendix A), adapted through focus group discussions from 24 questions developed by Pulerwitz et al [37]. Although the Pulerwitz gender norms scale was originally administered to men only, on review it was considered an appropriate measure of gender norms for both sexes and therefore administered to both women and men [38]. For gender norms, the Cronbach’s alpha was 0.72 for women and 0.75 for men. HIV stigma was measured by a set of 24 questions (appendix A), adapted from Sayles et al.’s 28-item scale [39]. The questions assessed the individual’s perceived HIV stigma in the community and internalised HIV stigma (referred throughout the rest of the paper as stigma). For stigma, the Cronbach’s alpha was 0.75 for women and 0.76 for men. An ART knowledge score was created by summing the number of ART-related questions answered correctly from a set of 8 questions developed for the study (appendix A). The physical violence questions were adapted from the physical assault scale of the ‘Revised Conflict Tactics Scale (CTS2)’ [40], and the social support from their partner was measured as a marker of ‘relationship quality’ using a set of 10 questions adapted from the 24-item Social Provision Scale (appendix A) [41]. The relationship quality scale was asked for each of the participant’s main partners. Participants with multiple partners were asked about the quality of each relationship for all partners they considered main partners. Among the 33 partnerships reported by participants with multiple partners, 11 (33%) partnerships did not have a relationship quality score because participants did not consider them main partnerships. The questionnaire was administered in a private room by study staff while participants waited to see ART clinic staff.

The questionnaire was translated into isiZulu, and reverse-translated to English independently to ensure integrity. In formative focus group discussions, it proved difficult in isiZulu to distinguish between answer options for some of the study questions, specifically between ‘strongly agree’ and ‘agree’ and similarly, ‘strongly disagree’ and ‘disagree’. For this reason, response options were limited to ‘agree’, ‘no opinion’, or disagree’ for questions with likert-scale answers.

Ethics approval of the project was given by the Biomedical Research Ethics Committee at the University of KwaZulu-Natal, South Africa, and the London School of Hygiene and Tropical Medicine, and permission to conduct the study in government clinics was granted by the Provincial Department of Health in Pietermaritzburg, KwaZulu-Natal, South Africa. Individuals were enrolled after giving written informed consent.

### Analysis

Study participants who reported having had sex in the year before enrolment were eligible for this analysis. Condom use at last sex within each partnership was measured in response to the question “Did you use a condom the last time you had sex with that partner”. Condom use referred to male or female condom use. However, female condom use in this area is rare [42].

Scores for gender norms, ART knowledge, stigma and relationship quality were calculated by assigning a value of three for the answer ‘agree’, two for ‘no opinion’, and one for ‘disagree’ for questions that were designed to be affirmative, the reverse for non-affirmative questions. For gender norms, higher scores indicate more equitable norms and lower scores indicate male dominant norms. The highest score possible was 57; median score was 41, interquartile range 37 to 45. Initially, indicator variables representing gender norms quartiles were considered, but the upper two quartiles and lower two quartiles were not significantly different from each other in their estimated association with condom use at last sex (data not shown). Thus, a binary variable of gender norms, categorising scores of ≤41 vs. ≥ 42 was used in the models. Similarly, a higher stigma score represents greater HIV-related stigma. The maximum stigma score was 72, median score 42, interquartile range 24 to 62. Stigma was considered in the models as a binary variable, representing scores of ≥42 as greater stigma vs. scores of ≤41. Approximately half the study participants (51.7%) answered all the ART knowledge questions correctly, achieving the maximum score of 24. Thus, a binary indicator of ART knowledge, representing scores of 24 vs. ≤23 was used in the analyses. A higher relationship quality score represents greater social support within the relationship. The maximum relationship quality score was 30, median score 24, an interquartile range 22 to 28. A binary indicator of relationship quality, representing scores of ≥25 vs. ≤24 was used in the analyses. Participant age, age at first sex, time since HIV diagnosis, length of partnership, and partner age difference were assessed categorically. Partner age difference was calculated by subtracting the woman’s age from the man’s age. Some participants were not able to report whether a partner was older or younger; others were, but did not know by how many years. Only the 368 women and 168 men who reported partner age differences are included in the categorical variable of age difference.

We hypothesized that condom use at last sex would be positively associated with more equitable gender norms among both women and men, adjusting for other potential confounders of condom use and gender norms. All analyses were sex-specific. Associations with p-values less than 0.05 were considered significant. We initially compared participant-level and partnership-level characteristics according to the two groups defined by condom use at last sex, and across gender norm groups (male dominant vs. more equitable gender norms) using Chi-square tests for categorical variables and Wilcoxon rank sum tests for continuous variables. When examining participant-level characteristics, condom use at last sex for participants in multiple relationships was considered to be ‘yes’ if a condom was used at last sex in any partnership. A sensitivity analysis of the Chi-square associations of condom use at last sex was conducted by removing participants who responded differently for condom use at last sex for different partners.

Given that condom use may vary by partnership, logistic regression models of partnerships for women and men were used to explore participant- and partnership-level factors associated with condom use at last sex. The models included variance adjustment for correlation between participants’ multiple ongoing partnerships using robust standard errors.

A priori, we hypothesized that age may modify the association between gender norms and condom use at last sex for both women and men [26,43]. Thus we initially tested for evidence of effect modification by including an interaction term between gender norms and age in logistic regression models of condom use at last sex that only included age and gender norms for each sex separately. Once effect modification by age had been established among women, age, gender norms, and their interaction were included in all multivariable models considered for women. This was not necessary for men given the lack of significant interactions. Factors identified in descriptive analysis as potential confounders of our association of interest or factors associated with the outcome were considered in the multivariable analyses. Wald tests were used to determine which variables remained in the final multivariable models.

Additional analyses focused on relationship quality, conflict, and fertility intentions as potential confounders of the association of interest using the subset of partnerships that were ongoing at baseline. All analyses were conducted using Stata, version 11.2 (StataCorp, College Station, Texas, USA).

## Results

Among the 632 participants, 530 reported having had one or more sexual partners in the year pre-enrolment, for a total of 550 partnerships. The median partnership length was 5 years (IQR 2–10); median age difference within partnerships was 4 (IQR 1–7) for women and -4 (IQR -8-0) for men; condoms were used at last sex in 58% of partnerships. Seventy-two (of 530) participants reported to no longer be in a partnership at baseline, 418 were in monogamous sexually-active partnerships, 26 in an ongoing but not currently sexually active (participant-defined) partnership, and 14 reported being sexually active with more than one partner. Of the participants reporting more than one partnership, two were women and 12 were men. Of these 14 participants, four responded differently for condom use at last sex for their different partners. One of these four participants was a woman; the other three participants were men. In sensitivity analysis that dropped these four participants, the estimated associations of participant level characteristics with condom use at last sex, results did not materially change.

Table 1 shows participant characteristics stratified by sex. Overall, 372 participants were women (70%), median age was 33 years (IQR 27–40): 31.5 years (IQR 26–38) among women, 37 years (IQR 31–44) among men (p<0.001). In addition to being statistically significantly older, men were less likely to have achieved secondary school or higher (56% vs. 71%), more likely to be ART-eligible (80% vs. 52%), be currently employed (34% vs. 19%), not have a current partner (18% vs. 12%), not always have lived within the ACDSA (65% vs. 51%), and have learned their HIV-positive status within the last year (78% vs. 52%), than women. The proportion with higher stigma did not differ significantly between women and men (p = 0.61), nor did the proportion with more equitable gender norms (p = 0.18). The reliability of the scales for stigma and gender norms for both sexes was good: for stigma, the Cronbach’s alpha was 0.75 for women and 0.76 for men and for gender norms, 0.72 for women and 0.75 for men.

**Table 1:** Participant characteristics by sex (N = 530).

Participant characteristic	Women N = 372 n (% of N)	Men N = 158 n (% of N)	p^a^
**Age (years)**			<0.001
17–25	82 (22)	13 (8)	
26–35	163 (44)	56 (35)	
36–45	91 (24)	55 (35)	
46+	36 (10)	34 (22)	
**ART eligibility group**			<0.001
ART-eligible	195 (52)	126 (80)	
Not yet ART-eligible	177 (48)	32 (20)	
**Gender norms**			0.18
Male dominant	226 (61)	86 (54)	
Equitable	146 (39)	72 (46)	
**Education**			0.008
<1 year	15 (4)	10 (6)	
Primary school	72 (19)	45 (28)	
Secondary, not matric	153 (41)	53 (34)	
Matric & higher	112 (30)	34 (22)	
Unknown	20 (5)	16 (10)	
**Employment status**			<0.001
Unemployed	300 (81)	104 (66)	
Employed	72 (19)	54 (34)	
**Partnership status**			<0.001
No current partnership	44 (12)	28 (18)	
Partnership but not sexually active^b^	16 (4)	10 (6)	
Sexually active^b^, 1 partner	310 (83)	108 (68)	
Sexually active^b^, multiple partners	2 (0.5)	12 (8)	
**Residential history** ^c^			0.01
Not always resident	152 (51)	72 (65)	
Always resident	144 (49)	38 (35)	
**Residential location** ^d^			0.76
Rural	140 (45)	53 (44)	
Urban / peri-urban	168 (55)	68 (56)	
**Internalised and perceived community stigma associated with HIV**			0.61
Low	195 (52)	79 (50)	
High	177 (48)	79 (50)	
**ART Knowledge**			0.20
Low	173 (47)	83 (53)	
High	199 (53)	75 (47)	
**Time (prior to baseline) since HIV diagnosis**			<0.001
< 1 year	194 (52)	123 (78)	
≥ 1 year	178 (48)	35 (22)	

^a^Chi-square test.

^b^Sexually active / not sexually active is participant-defined.

^c^Available for N = 406. In the Africa Centre surveillance system household membership is not conditional on residency, an individual can be recorded as a non-resident household member if they are residing in a household outside the demographic surveillance area (DSA) but remain socially connected to a household in the DSA. Changes in residence by individuals are documented within the DSA and into or out of the DSA since January 2000.

^d^Available for N = 429. Households reside in a location that is designated rural if population density<400 per km^2^.

Education was positively associated with more equitable gender norms among both women (p<0.001) and men (p = 0.002), while age and ART knowledge were negatively associated with more equitable gender norms (Table 2). Stigma (p = 0.003) and time since HIV diagnosis (p = 0.003) were negatively associated with condom use at last sex among women. More equitable gender norms were positively associated with condom use at last sex among men (p = 0.02), but not women (p = 0.27). Higher education (p = 0.01), being currently employed (p = 0.002), and currently having a partner (p = 0.02) were also positively associated with condom use at last sex among men. Among both sexes, ART-eligibility (measured by CD4 count and/or clinical status) was not associated with condom use at last sex and thus not a potential confounder of the association between gender norms and condom use (women p = 0.24, men p = 0.82).

**Table 2:** The association of participant characteristics with gender norms and condom use at last sex, stratified by sex (N = 530).

Participant characteristic	Women with equitable gender norms (%)	p^a^	Men with equitable gender norms (%)	p^a^	Women who used a condom at last sex (%)^b^	p^a^	Men who used a condom at last sex (%)^b^	p^a^
**Age (years)**		<0.001		0.04		0.02		0.03
17–25	42 (51)		10 (77)		41 (50)		10 (77)	
26–35	73 (45)		28 (50)		102 (63)		31 (55)	
36–45	23 (25)		23 (42)		55 (60)		40 (73)	
46+	8 (22)		11 (32)		13 (36)		15 (44)	
**ART eligibility group**		0.11		0.15		0.24		0.82
ART-eligible	84 (43)		61 (48)		105 (54)		76 (60)	
Not yet ART-eligible	62 (35)		11 (34)		106 (60)		20 (63)	
**Gender norms**						0.27		0.02
Male dominant					123 (54)		45 (52)	
Equitable					88 (60)		51 (71)	
**Education**		<0.001		0.002		0.13		0.01
<1 year	1 (7)		2 (20)		8 (53)		5 (50)	
Primary school	15 (21)		13 (29)		33 (46)		22 (49)	
Secondary, not matric	60 (39)		26 (49)		87 (57)		36 (68)	
Matric & higher	68 (61)		24 (71)		73 (65)		27 (79)	
Unknown	2 (10)		7 (44)		10 (50)		6 (38)	
**Employment status**		0.64		0.90		0.40		0.002
Unemployed	116 (39)		47 (45)		167 (56)		54 (52)	
Employed	30 (42)		25 (46)		44 (61)		42 (78)	
**Partnership status**		0.007		0.67		0.003		0.02
No current partnership	21 (48)		14 (50)		15 (34)		10 (36)	
Partnership but not sexually active^c^	12 (75)		6 (60)		6 (38)		6 (60)	
Sexually active^c^, 1 partner	113 (36)		46 (43)		189 (61)		73 (68)	
Sexually active^c^, multiple partners	0 (0)		6 (50)		1 (50)		7 (58)	
**Residential history** ^d^		0.21		0.23		0.18		0.45
Not always resident	70 (46)		39 (54)		92 (61)		42 (58)	
Always resident	56 (39)		16 (42)		76 (53)		25 (66)	
**Residential location** ^e^		0.34		0.20		0.49		0.26
Rural	55 (39)		21 (40)		83 (59)		35 (66)	
Urban / peri-urban	75 (45)		35 (51)		93 (55)		38 (56)	
**Internalised and perceived community stigma associated with HIV**		0.77		0.80		0.003		0.46
Low	63 (38)		32 (44)		107 (65)		46 (64)	
High	83 (40)		40 (47)		104 (50)		50 (58)	
**ART Knowledge**		<0.001		<0.001		0.66		0.85
Low	89 (51)		50 (60)		96 (55)		51 (61)	
High	57 (29)		22 (29)		115 (58)		45 (60)	
**Time (prior to baseline) since HIV diagnosis**		0.98		0.72		0.003		0.50
<1 year	70 (39)		15 (43)		115 (65)		23 (66)	
≥ 1 year	76 (39)		57 (46)		96 (49)		73 (59)	

^a^Chi-square test.

^b^Condom use at last sex for participants in multiple relationships was considered to be ‘yes’ if a condom was used at last sex in any partnership.

^c^Sexually active / not sexually active is participant-defined.

^d^Available for N = 406. In the Africa Centre surveillance system household membership is not conditional on residency, an individual can be recorded as a non-resident household member if they are residing in a household outside the demographic surveillance area (DSA) but remain socially connected to a household in the DSA. Changes in residence by individuals are documented within the DSA and into or out of the DSA since January 2000.

^e^Available for N = 429. Households reside in a location that is designated rural if population density<400 per km^2^.

Table 3 stratifies partnership characteristics by sex. Women were significantly more likely than men to report alcohol use at the time of last sex by them or their partner (14% vs. 11%), have a partner older than themselves (92% vs. 18%), have had their partner insist on having sex when they didn’t want to (27% vs. 13%), have ever refused to have sex with their partner (50% vs.18%), believe their partner had sex with someone else in the last six months (46% vs. 10%), have a currently employed partner (71% vs. 24%), and not be living with their partner (50% vs. 39%). There was no significant difference by sex in the proportion that had disclosed their HIV status to their partner (p = 0.55). However, significantly more men than women reported knowing their partner’s HIV status (50% vs. 36%). Within partnerships, condom use (ever, at first sex, and at last sex) did not significantly differ between women and men.

**Table 3:** Partnership characteristics by sex (N = 550).

Partnership characteristic	Women N = 375 n (% of N)	Men N = 175 n (% of N)	p^a^
**Condom use at last sex**			0.37
No	161 (43)	68 (39)	
Yes	214 (57)	107 (61)	
**Partnership length (years)**			0.03
<1	21 (6)	16 (9)	
1–2	94 (25)	25 (14)	
3–5	91 (24)	54 (31)	
6–9	67 (18)	28 (16)	
10+	102 (27)	52 (30)	
**Ever used a condom with partner**			0.71
No	89 (24)	39 (22)	
Yes	286 (76)	136 (78)	
**Condom use at first sex with partner**			0.07
No	307 (82)	154 (88)	
Yes	68 (18)	21 (12)	
**Partner living arrangement**			0.006
Lives with participant	148 (39)	91 (52)	
Does not live with participant	227 (61)	84 (48)	
**Alcohol involved at last sex**			0.009
No	323 (86)	164 (94)	
Yes	52 (14)	11 (6)	
**Partner currently employed**			<0.001
No	108 (29)	133 (76)	
Yes	266 (71)	42 (24)	
Don’t Know	1 (0.3)	0 (0)	
**Partner Age Difference** ^b^ **(years)**			<0.001
≤ -1	29 (8)	121 (72)	
0–2	94 (26)	31 (18)	
3–5	113 (31)	8 (5)	
6+	132 (36)	8 (5)	
**Partner ever insist to have sex when you don’t want to?**			<0.001
Never	273 (73)	153 (87)	
Sometimes / Often	102 (27)	22 (13)	
**Ever refuse to have sex with this partner?**			<0.001
No	186 (50)	144 (82)	
Yes	189 (50)	31 (18)	
**Partner had sex with other people in the last 6 months?**			<0.001
No	100 (27)	87 (50)	
Don't Know	101 (27)	69 (39)	
Yes, I think so	35 (9)	6 (3)	
Yes, definitely	139 (37)	13 (7)	
**Disclosed HIV status to partner**			0.55
No	132 (35)	57 (33)	
Yes	243 (65)	118 (67)	
**Knowledge of partner’s HIV status**			0.01
Knows s/he is HIV+	118 (31)	75 (43)	
Knows s/he is HIV-	18 (5)	12 (7)	
Does not know status	239 (64)	88 (50)	
**Among ongoing main partnerships (N = 467)**			
**Recently argued**			0.29
No	222 (67)	99 (72)	
Yes	108 (33)	38 (28)	
**Ever used physical violence towards partner**			<0.001
No	307 (93)	110 (80)	
Yes	23 (7)	27 (20)	
**Partner ever used physical violence towards participant**			0.003
No	284 (86)	131 (96)	
Yes	46 (14)	6 (4)	
**Relationship quality** ^**c**^			0.01
Lower quality	178 (54)	57 (42)	
Higher quality	151 (46)	80 (58)	
**Wishes to have more children with partner**			0.03
No	231 (70)	82 (60)	
Yes	99 (30)	55 (40)	

^a^Chi-square test.

^b^Woman's age subtracted from man's age. Available for N = 368 women N = 168 men.

^c^Available for N = 466.

Table 4 shows partnership characteristics of women and men across gender norms groups and between groups defined by condom use at last sex. Among partnerships reported by women, condom use ever (p = 0.001) and at first sex (p<0.001), not disclosing HIV status to partner (p = 0.03), and knowing partner is HIV-negative (p = 0.003) were positively associated with more equitable gender norms. Condom use ever (p<0.001) and at first sex (p<0.001), no alcohol use at last sex (p = 0.04), never having unwanted sex with partner (p = 0.03), disclosure of HIV status to partner (p<0.001), and knowledge of partner’s HIV status (p<0.001) were positively associated with condom use at last sex for women. Condom use at last sex among women was negatively associated with reports that their partner had definitely had sex with someone else in the last six months (p = 0.04). Partner age difference was not significantly associated with condom use at last sex among either sex.

**Table 4:** Partnership characteristics associated with equitable gender norms and condom use at last sex (N = 550).

Partnership characteristic	Women with equitable gender norms (%)	p^a^	Men with equitable gender norms (%)	p^a^	Women who used a condom at last sex (%)	p^a^	Men who used a condom at last sex (%)	p^a^
**Condom use at last sex**		0.32		0.008				
No	58 (36)		23 (34)		−−−−		−−−−	
Yes	88 (41)		58 (54)		−−−−		−−−−	
**Partnership length (years)**		0.30		0.73		0.67		0.82
<1	10 (48)		5 (31)		11 (52)		9 (56)	
1–2	40 (43)		12 (48)		53 (56)		13 (52)	
3–5	36 (40)		27 (50)		53 (58)		33 (61)	
6–9	29 (43)		14 (50)		43 (64)		18 (64)	
10+	31 (31)		23 (44)		54 (53)		34 (65)	
**Ever used a condom with partner**		0.001		0.003		<0.001		<0.001
No	21 (24)		10 (26)		0 (0)		0 (0)	
Yes	125 (44)		71 (52)		214 (75)		107 (79)	
**Condom use at first sex with partner**		<0.001		0.001		<0.001		0.13
No	106 (35)		64 (42)		159 (52)		91 (59)	
Yes	40 (59)		17 (81)		55 (81)		16 (76)	
**Partner living arrangement**		<0.001		0.34		0.59		0.18
Lives with participant	40 (27)		39 (43)		87 (59)		60 (66)	
Does not live with participant	106 (47)		42 (50)		127 (56)		47 (56)	
**Alcohol involved at last sex**		0.19		0.05		0.04		0.08
No	130 (40)		79 (48)		191 (59)		103 (63)	
Yes	16 (31)		2 (18)		23 (44)		4 (37)	
**Partner currently employed**		0.24		0.36		0.47		0.91
No	37 (34)		59 (44)		60 (56)		81 (61)	
Yes	108 (41)		22 (52)		154 (58)		26 (62)	
Don’t Know	1 (100)		−−−−		0 (0)		−−−−	
**Partner Age Difference** ^b^ **(years)**		0.16		0.76		0.57		0.42
≤ -1	7 (24)		54 (45)		13 (45)		79 (65)	
0–2	42 (45)		13 (42)		53 (56)		18 (58)	
3–5	48 (42)		5 (63)		65 (57)		5 (63)	
6+	47 (36)		5 50)		78 (59)		3 (38)	
**Partner ever insist to have sex when you don’t want to?**		0.31		0.32		0.03		0.50
Never	102 (37)		73 (48)		165 (60)		95 (62)	
Sometimes / Often	44 (43)		8 (36)		49 (48)		12 (55)	
**Ever refuse to have sex with this partner?**		0.93		0.59		0.39		0.23
No	72 (39)		68 (47)		102 (55)		91 (63)	
Yes	74 (39)		13 (42)		112 (59)		16 (52)	
**Partner had sex with other people in the last 6 months?**		0.05		<0.001		0.04		0.60
No	33 (33)		26 (30)		62 (62)		52 (60)	
Don't Know	44 (44)		46 (67)		66 (65)		45 (65)	
Yes, I think so	20 (57)		4 (67)		16 (46)		4 (67)	
Yes, definitely	49 (35)		5 (38)		70 (50)		6 (46)	
**Disclosed HIV status to partner**		0.03		0.84		<0.001		<0.001
No	61 (46)		27 (47)		48 (36)		24 (42)	
Yes	85 (35)		54 (46)		166 (68)		83 (70)	
**Knowledge of partner’s HIV status**		0.003		0.71		<0.001		0.003
Knows s/he is HIV+	31 (26)		32 (43)		88 (75)		56 (75)	
Knows s/he is HIV-	9 (50)		6 (50)		13 (72)		8 (67)	
Does not know status	146 (39)		43 (49)		113 (47)		43 (49)	
**Among ongoing partnerships (N = 467)**								
**Recently argued**		0.002		0.03		0.96		0.01
No	71 (32)		39 (39)		133 (60)		61 (62)	
Yes	54 (50)		23 (61)		65 (60)		32 (84)	
**Ever used physical violence towards partner**		0.14		0.23		0.93		0.03
No	113 (37)		47 (43)		184 (60)		70 (64)	
Yes	12 (52)		15 (56)		14 (61)		23 (85)	
**Partner ever used physical violence towards participant**		0.89		0.81		0.27		0.34
No	108 (38)		59 (45)		167 (59)		90 (69)	
Yes	17 (37)		3 (50)		31 (67)		3 (50)	
**Relationship quality** ^**c**^		0.12		0.44		<0.001		0.50
Lower quality	74 (42)		24 (42)		91 (51)		36 (63)	
Higher quality	50 (33)		29 (49)		107 (71)		55 (69)	
**Wishes to have more children with partner**		0.54		0.17		0.88		0.62
No	85 (37)		41 (50)		138 (60)		57 (70)	
Yes	40 (40)		21 (38)		60 (61)		36 (65)	

^a^Chi-square test.

^b^Woman's age subtracted from man's age. Available for N = 368 women N = 168 men.

^c^Available for N = 466.

Among partnerships reported by men, condom use ever (p = 0.003), at first sex (p = 0.001), and at last sex (p = 0.008), and no alcohol use at last sex (p = 0.05) were positively associated with more equitable norms. Condom use at last sex was positively associated with ever using a condom within the partnership (p<0.001), disclosure of HIV status to partner (p<0.001), and knowledge of partner’s HIV status (p = 0.003) for men.

In models that included age, gender norms and their interaction, age was found to be an effect modifier of the association between gender norms and condom use at last sex for women (Wald p = 0.01), but not for men (Wald p = 0.90). In the final multivariable model for women, being sexually active with their current partner and disclosure of HIV status to their partner remained significantly associated with increased odds of a condom being used at last sex, while having higher levels of stigma, having unwanted sex with partner, and not knowing partner’s HIV status remained associated with significantly lower odds of condom use at last sex (Table 5). The interaction between age and gender norms remained significant in the final logistic regression model for women. However, the only significant odds ration (OR) estimate from the interaction was for women <26 years with equitable gender norms compared to the same age group with male dominant gender norms, (OR 8.88, 95% CI 2.95–26.75). Table 5 also reports the univariate OR estimates for the variables that remained in the final model and shows little confounding between the variables included in the final model. The multivariable model was repeated excluding participants with multiple partners and all estimates, including effect modification by age, remained similar to the final model.

**Table 5:** Logistic regression models for odds of condom use at last sex^a^.

Characteristic	Univariate Model				Multivariable Model^b^				Univariate Model				Multivariable Model^c^		
	Odds Ratio	95% CI	p^d^		Odds Ratio	95% CI	p^d^		Odds Ratio	95% CI	p^d^		Odds Ratio	95% CI	p^d^
**Gender norms**	** **	** **	** **	** **	** **	** **	** **	** **			0.01				0.10
Male dominant									1.00				1.00		
Equitable									2.32	1.19, 4.52			1.90	0.89, 4.10	
**Gender norms by age**			0.01				0.005				0.90				
Among 17–25: Male dominant	1.00				1.00				1.00						
Equitable	3.60	1.47, 8.85			8.88	2.95, 26.75			1.50	0.08, 27.55					
Among 26–35: Male dominant	1.00				1.00				1.00						
Equitable	1.13	0.59, 2.15			2.00	0.97, 4.09			2.70	0.95, 7.70					
Among 36–45: Male dominant	1.00				1.00				1.00						
Equitable	0.39	0.15, 1.03			0.52	0.16, 1.65			1.65	0.50, 5.43					
Among 46+: Male dominant	1.00				1.00				1.00						
Equitable	1.08	0.21, 5.51			1.37	0.30, 6.21			3.00	0.58, 15.46					
**Education**															0.02
<1 year									1.00				1.00		
Primary school									0.88	0.22, 3.49			0.57	0.15, 2.16	
Secondary, not matric									2.32	0.59, 9.17			1.40	0.35, 5.57	
Matric & higher									3.33	0.76, 14.54			2.45	0.64, 9.33	
Unknown									0.60	0.12, 2.99			0.44	0.09, 2.07	
**Employment Status**											0.001				0.001
Unemployed									1.00				1.00		
Employed									3.44	1.65, 7.19			3.20	1.49, 6.87	
**Partnership status**			0.002				<0.001								
No current partnership	1.00				1.00										
Partner but not sexually active^e^	1.16	0.35, 3.81			0.61	0.17, 2.15									
Sexually active^e^, 1 partner	3.02	1.55, 5.87			2.37	1.14, 4.93									
Sexually active^e^, multiple partners	7.73	1.69, 35.42			46.52	15.09, 143.41									
**Internalised and perceived community stigma associated with HIV**			0.003				0.02								
Low	1.00				1.00										
High	0.54	0.36, 0.81			0.55	0.34, 0.89									
**Partner ever insist to have sex when you don’t want to?**			0.03				0.01								
Never	1.00				1.00										
Sometimes / Often	0.61	0.38, 0.96			0.51	0.30, 0.88									
**Disclosed HIV status to partner**			<0.001				<0.001				<0.001				0.001
No	1.00				1.00				1.00				1.00		
Yes	3.77	2.41, 5.91			2.86	1.64, 4.96			0.68	0.19, 2.43			3.85	1.69, 8.75	
**Knowledge of partner’s HIV status**			<0.001				0.01								
Knows s/he is HIV+	1.00				1.00										
Knows s/he is HIV-	0.89	0.29, 2.70			1.19	0.31, 4.62									
Does not know status	0.31	0.19, 0.50			0.42	0.23, 0.76		** **							

^a^Adjusted for partnership clustering using robust standard errors.

^b^The following variables did not contribute to the multivariable model for women: Knowledge of partner having sex with others in last 6 months and alcohol use at last sex. Relationship quality was not significant when added to the model.

^c^The following variables did not contribute to the multivariable model for men: partnership status and knowledge of partner's HIV status. Having recently argued or ever used physical violence towards partner were not significant in univariate models.

^d^Wald test. In the case of equitable gender norms by age, the Wald p-value is for the interaction term added to a model with gender norms and age as main effects.

^e^Sexually active / not sexually active is participant-defined.

In the final multivariable model for men, currently employed and disclosure of HIV status to their partner remained significantly associated with increased odds of condom use at last sex (Table 5). Education also remained in the model as a confounder. Gender norms were not significantly associated with condom use at last sex among men in the final multivariable model. Univariate OR estimates are also shown for the variables that remained in the final model (Table 5).

In the subset of main partnerships ongoing at baseline (N = 467, Table 4), women were significantly more likely than men to have had their partner ever use physical violence against them (14% vs. 4%) and to report lower relationship quality (58% vs. 46%); men were significantly more likely than women to have used physical violence against their partner (20% vs. 7%) and to want more children with their partner (40% vs. 30%). Among women, higher relationship quality was positively associated with condom use at last sex, but did not remain significant when added to the final multivariable model for women (Table 5). The reliability of the relationship scale for both sexes was good. The Cronbach’s alpha for relationship quality was 0.76 for women and 0.74 for men. Among men, having recently argued (p = 0.01) and having ever used physical violence towards a partner (p = 0.03) were positively associated with condom use. However, neither variable provided significant additional contributions to the multivariable model for men in Table 5.

## Discussion

We found younger HIV-positive women reporting more equitable gender norms were significantly more likely to have used a condom at last sex than those with male dominant gender norms. Although the association between equitable gender norms and condom use is clear for women aged 25 or below it was less so in the older age groups of women and men of all ages. The lack of an association between gender norms and condom use in men and the over 25 year old women may reflect a difference in the way HIV-positive men and older women negotiate condom use.

While gender inequality is a recognised important driver of the HIV epidemic in Africa [44], few studies have measured its association with condom use among HIV-positive women and men. One intervention study in South Africa found that, irrespective of HIV status, condom use was significantly associated with condom negotiation, and condom use with a primary partner increased among women participating in a woman-focused HIV intervention [45]. A study of women aged 18–49 years in Botswana and KwaZulu-Natal used a large age difference within partnerships as a proxy for gender power imbalance. Condom use was associated with gender power imbalance for both sexes; women with partners more than 10 years older were less able to suggest using condoms to their partners, and men were more likely to refuse condoms when the age difference within a partnership was large [46]. This study did not adjust for participant age. In our cohort, very few (N = 75) partners had an age difference of 10 years or more. An evaluation of the Stepping Stones gender-focused project involving young adults aged 15–26 years did not observe any change in condom use at last sex among women or men in the intervention compared to the control group [47]. Identifying the key elements of gender transformative interventions remains critically important in terms of supporting positive prevention strategies.

Just over half of the participants in our study reported using a condom at their last sex act, consistent with data on condom use with regular partners from the population-based surveillance programme in the same area [13], and data collected among rural HIV-positive adults elsewhere in KwaZulu-Natal [7]. However, reported condom use in this cohort is lower than among HIV-positive individuals seeking treatment in urban areas of South Africa [9–11].

Our data demonstrates a negative association between condom use and previous experience of unwanted sex among women, consistent with literature on the association between intimate partner violence and low condom use in South Africa [48]. Stigma was also negatively associated with condom use among women. Interestingly, women’s experience of stigma was no higher than men’s in this cohort, in contrast to reports from other countries [49]. In Kenya, Mugoya et al. found that HIV-related knowledge was significantly inversely associated with stigma levels for both women and men; and it is possible that the lack of significant sex differences in stigma scores for our study participants reflects a high level of HIV-related knowledge.

Consistent with literature that has identified higher socioeconomic status as a predictor of increased condom use in KwaZulu-Natal, employment was a strong predictor of condom use for men [50]. Education was an important confounder of the relationship between gender norms and condom use among men, suggesting that education is key to reducing gender inequity and preventing HIV in rural South Africa. In contrast to previous studies, having recently argued and ever use of physical violence towards a partner were positively associated with condom use among the HIV-positive men in ongoing partnerships [23, 47]. Further exploration is needed to determine if this finding is an artefact of the data.

Our findings demonstrate that both HIV-positive women and men who disclosed their status to their partner or who knew their partner’s HIV status were significantly more likely to use a condom at last sex [7,9,10,29]. Interestingly, whereas the partner’s HIV status was not significantly associated with condom use, knowledge of partner’s HIV status was important, suggesting that communication between partners plays a critical role in determining condom use. It is encouraging that the majority of participants had disclosed their HIV status to their partner, but discouraging that the majority did not know their partners’ status, typically because their partner had not tested for HIV. Interestingly, we found no association between a history of migration and condom use, nor between place of residence and condom use, despite reported condom use usually being higher among urban residents [51].

The proportion of women and men enrolled in the study are broadly representative of women and men attending the local ART programme and the sex-ratio of those on ART and in pre-ART care[13]. The trend of men accessing ART at much lower rates than women in South Africa [52] may reflect earlier diagnosis through antenatal testing among women as well as men avoiding treatment to prevent appearing ‘weak’ [52,53].

Strengths of this study include the cohort design, range of socio-behavioural variables that uniquely capture the behaviour and attitudes of HIV-positive individuals in a region of high HIV prevalence, large sample size, and sex-stratified analysis. In addition, the cohort is broadly representative of individuals with CD4>500 cells/ml and treatment-initiators in the local ART programme [36].

There are limitations to be considered when interpreting our results. Participants were not randomly selected: they were individuals who knew their HIV status, had chosen to seek care, and agreed to participate in a cohort study designed to assess sexual behaviour among HIV-positive adults. Although the ART eligible groups in our cohort were similar in age and sex distribution to the local ART programme [36], we need to be cautious in generalising our findings to all HIV-positive women and men. It is possible that women and men attending the ART programme may be more likely to have equitable gender norms than HIV-positive women and men not in the programme. Men accessing the clinic may be more likely to have equitable gender norms than men not accessing the clinic because they may be less likely to feel that their masculinity is threatened by seeking assistance than men not accessing the clinic. Women accessing the clinic may be more empowered and therefore also have more equitable gender norms than their counterparts not accessing the clinic. In addition, all data were self-reported, and thus susceptible to social desirability bias [54]. We would expect that social desirability would have less of an effect on reporting among adults who volunteered to participate in this cohort. However, it is unclear whether social desirability bias would impact differentially for individuals with equitable or male dominant gender norms.

It is important to note that there is no gold standard for measuring condom use [55,56]; reported condom use at last sex is more accurate than estimating the rate of condom use in the last month [54]. Condom use at last sex act has also been found to be a reasonable indicator of condom use in the last week [57]. However, condom use at last sex act may be a poor indicator of consistent condom use over time [58].

## Conclusions

This study highlights the importance of gender equality for condom use among young HIV-positive women in KwaZulu-Natal and the need to more consistently collect data on gender equity in studies evaluating sexual behaviour. It also illustrates the need to promote communication about HIV status between couples and challenge HIV-related stigma. Strategies to address gender equity should be integrated into positive prevention interventions targeting both women and men, and supported by efforts at a societal level to decrease gender inequality.

## References

[1] UNAIDS (2013).

[2] Zaidi J, Grapsa E, Tanser F, Newell ML, Barnighausen T (2013) Dramatic increases in HIV prevalence after scale-up of antiretroviral treatment: a longitudinal population-based HIV surveillance study in rural kwazulu-natal. AIDS. 10.1097/QAD.0b013e328362e832 PMC4264533 23669155

[3] Cohen MS, Chen YQ, McCauley M, Gamble T, Hosseinipour MC, Nagalingeswaran K, et al (2011) Prevention of HIV-1 infection with early antiretroviral therapy. N Engl J Med 365: 493–505. 10.1056/NEJMoa1105243 21767103 PMC3200068

[4] The South African Department of Health (2013) The South African Antiretroviral Treatment Guidelines, 2013.

[5] (2014) Health budget vote speech by the Minister of Health, Dr. Aaron Motsoaledi, MP. Department of Health, Republic of South Africa.

[6] South African National AIDS Council (2012) Global AIDS response progress report.

[7] Lurie M, Pronyk P, de Moor E, Heyer A, de Bruyn G, Struthers H, et al (2008) Sexual behavior and reproductive health among HIV-infected patients in urban and rural South Africa. JAcquirImmuneDeficSyndr 47: 484–493. 10.1097/QAI.0b013e3181648de8 PMC3811008 18209685

[8] Wong LH, Rooyen HV, Modiba P, Richter L, Gray G, McIntyre J, et al (2009) Test and tell: correlates and consequences of testing and disclosure of HIV status in South Africa (HPTN 043 Project Accept). J Acquir Immune Defic Syndr 50: 215–222. 10.1097/QAI.0b013e3181900172 19131885 PMC2729272

[9] Kiene SM, Christie S, Cornman DH, Fisher WA, Shuper PA, Pillay S, et al (2006) Sexual risk behaviour among HIV-positive individuals in clinical care in urban KwaZulu-Natal, South Africa. AIDS 20: 1781–1784. 16931945 10.1097/01.aids.0000242827.05120.55

[10] Venkatesh KK, de Bruyn G, Lurie MN, Modisenyane T, Triche EW, Gray GE, et al (2012) Sexual risk behaviors among HIV-infected South African men and women with their partners in a primary care program: implications for couples-based prevention. AIDS Behav 16: 139–150. 10.1007/s10461-011-9941-y 21476005 PMC3184366

[11] Vu L, Andrinopoulos K, Mathews C, Chopra M, Kendall C, Eisele TP (2012) Disclosure of HIV status to sex partners among HIV-infected men and women in Cape Town, South Africa. AIDS Behav 16: 132–138. 10.1007/s10461-010-9873-y 21197600

[12] Shisana O, Simbayi L, Rehle T, Zungu N, Zuma K, Ngogo N (2010) South African national HIV prevalence incidence behaviour and communication survey 2008: the health of our children.

[13] McGrath N, Eaton JW, Barnighausen TW, Tanser F, Newell ML (2013) Sexual behaviour in a rural high HIV prevalence South African community: time trends in the antiretroviral treatment era. AIDS. 10.1097/01.aids.0000432473.69250.19 PMC3773237 23842132

[14] Kalichman SC, Simbayi LC, Cain D (2010) HIV transmission risk behaviours among HIV seropositive sexually transmitted infection clinic patients in Cape Town, South Africa. Eur J Public Health 20: 202–206. 10.1093/eurpub/ckp127 19726591 PMC2860715

[15] Simbayi LC, Kalichman SC, Strebel A, Cloete A, Henda N, Mgeketo A (2007) Disclosure of HIV status to sex partners and sexual risk behaviours among HIV-positive men and women, Cape Town, South Africa. Sex TransmInfect 83: 29–34. 16790562 10.1136/sti.2006.019893 PMC2598581

[16] Eisele TP, Mathews C, Chopra M, Brown L, Silvestre E, Daries V et al (2008) High levels of risk behavior among people living with HIV Initiating and waiting to start antiretroviral therapy in Cape Town South Africa. AIDS Behav 12: 570–577. 17636372 10.1007/s10461-007-9279-7

[17] Eisele TP, Mathews C, Chopra M, Lurie MN, Brown L, Dewing S, et al (2009) Changes in risk behavior among HIV-positive patients during their first year of antiretroviral therapy in Cape Town South Africa. AIDS Behav 13: 1097–1105. 10.1007/s10461-008-9473-2 18846418

[18] Kaida A, Gray G, Bastos FI, Andia I, Maier M, McIntyre J, et al (2008) The relationship between HAART use and sexual activity among HIV-positive women of reproductive age in Brazil, South Africa, and Uganda. AIDS Care 20: 21–25. 10.1080/09540120701426540 18278611

[19] Olley B, Zeier M, Seedat S, Stein D (2005) Post-traumatic stress disorder among recently diagnosed patients with HIV/AIDS in South Africa. AIDS care 17: 550–557. 16036241 10.1080/09540120412331319741

[20] Rosenberg NE, Pettifor AE, De Bruyn G, Westreich D, Delany-Moretlwe S, Behets F, et al (2013) HIV testing and counseling leads to immediate consistent condom use among South African stable HIV-discordant couples. J Acquir Immune Defic Syndr 62: 226–233. 10.1097/QAI.0b013e31827971ca 23117500 PMC3548982

[21] Mantell JE, Smit JA, Beksinska M, Scorgie F, Milford C, Balch E, et al (2011) Everywhere you go, everyone is saying condom, condom. But are they being used consistently? Reflections of South African male students about male and female condom use. Health Educ Res 26: 859–871. 10.1093/her/cyr041 21693684 PMC3168335

[22] Dunkle KL, Jewkes RK, Brown HC, Gray GE, McIntryre JA, Harlow SD (2004) Gender-based violence, relationship power, and risk of HIV infection in women attending antenatal clinics in South Africa. Lancet 363: 1415–1421. 15121402 10.1016/S0140-6736(04)16098-4

[23] O'Sullivan LF, Harrison A, Morrell R, Monroe-Wise A, Kubeka M (2006) Gender dynamics in the primary sexual relationships of young rural South African women and men. Cult Health Sex 8: 99–113. 16641060 10.1080/13691050600665048

[24] Pettifor AE, Measham DM, Rees HV, Padian NS (2004) Sexual power and HIV risk, South Africa. EmergInfectDis 10: 1996–2004. 10.3201/eid1011.040252 PMC3328992 15550214

[25] Susser I (2009) AIDS, Sex and Culture: Global Politics and Survival in Southern Africa. UK: Wiley-Backwell.

[26] Ncube N (2010) Gender inequality and HIV risk in South Africa: Education and socio-economic empowerment of women as protective factors against HIV infection. The International Journal of Learning 16: 489–496.

[27] Hunter M (2010) Love in the Time of AIDS: Inequality, Gender, and Rights in South Africa. Bloomington, Indiana, USA: Indiana University Press.

[28] Pettifor A, Macphail C, Anderson AD, Maman S (2012) 'If I buy the Kellogg's then he should [buy] the milk': young women's perspectives on relationship dynamics, gender power and HIV risk in Johannesburg, South Africa. Cult Health Sex 14: 477–490. 10.1080/13691058.2012.667575 22449022 PMC3570821

[29] Mantell JE, Needham SL, Smit JA, Hoffman S, Cebekhulu Q, Adams-Skinner J et al (2009) Gender norms in South Africa: implications for HIV and pregnancy prevention among African and Indian women students at a South African tertiary institution. Cult Health Sex 11: 139–157. 10.1080/13691050802521155 19247859 PMC2782559

[30] Hunter M (2005) Cultural politics and masculinities: Multiple-partners in historical perspective in KwaZulu-Natal. CultHealth Sex 7: 389–403. 16864211 10.1080/13691050412331293458

[31] Varga CA (1997) Sexual decision-making and negotiation in the midst of AIDS: youth in KwaZulu-Natal, South Africa. Health TransitRev 7: 45–67.

[32] Jewkes R, Morrell R (2010) Gender and sexuality: emerging perspectives from the heterosexual epidemic in South Africa and implications for HIV risk and prevention. J Int AIDS Soc 13: 6 10.1186/1758-2652-13-6 20181124 PMC2828994

[33] MacPhail C, Terris-Prestholt F, Kumaranayake L, Ngoako P, Watts C, Rees H (2009) Managing men: women's dilemmas about overt and covert use of barrier methods for HIV prevention. CultHealth Sex 11: 485–497. 10.1080/13691050902803537 19479490

[34] Houlihan CF, Bland RM, Mutevedzi PC, Lessells RJ, Ndirangu J, Thulare H, et al. (2010) Cohort Profile: Hlabisa HIV Treatment and Care Programme. IntJ Epidemiol. 10.1093/ije/dyp402 PMC3195268 20154009

[35] McGrath N, Richter L, Newell ML (2011) Design and methods of a longitudinal study investigating the impact of antiretroviral treatment on the partnerships and sexual behaviour of HIV-infected individuals in rural KwaZulu-Natal, South Africa. BMC Public Health 11: 121 10.1186/1471-2458-11-121 21333022 PMC3049146

[36] McGrath N, Richter LR, Newell ML (2013) Sexual risk after HIV diagnosis: A comparison of pre-ART individuals with CD4>500 cells/μl and ART-eligible individuals in an HIV treatment and care programme in rural KwaZulu-Natal, South Africa. J Int AIDS Soc. 10.7448/IAS.16.1.18048 PMC3736456 23920209

[37] Pulerwitz J, Barker G (2008) Measuring attitudes towards gender norms among young men in Brazil. Development and psychometric evaluation of the GEM scale. Men and Masculinities 10: 322–338.

[38] Richter LR (2008).

[39] Sayles JN, Hays RD, Sarkisian CA, Mahajan AP, Spritzer KL, Cunningham WE (2008) Development and psychometric assessment of a multidimensional measure of internalized HIV stigma in a sample of HIV-positive adults. AIDS Behav 12: 748–758. 10.1007/s10461-008-9375-3 18389363 PMC2858334

[40] Straus M, Sugarman D (1996) The Revised Conflict Tactics Scales (CTS2). Journal of Family Issues 17: 283–316.

[41] Cutrona C, Russell D (1987) The provisions of social relationships and adaptation to stress. Advances in Personal Relationships: 37–67.

[42] Skoler-Karpoff S, Ramjee G, Ahmed K, Altini L, Plagianos MG, Friedland B, et al (2008) Efficacy of Carraguard for prevention of HIV infection in women in South Africa: a randomised, double-blind, placebo-controlled trial. Lancet 372: 1977–1987. 10.1016/S0140-6736(08)61842-5 19059048

[43] Chimbindi NZ, McGrath N, Herbst K, San Tint K, Newell ML (2010) Socio-Demographic Determinants of Condom Use Among Sexually Active Young Adults in Rural KwaZulu-Natal, South Africa. Open AIDS J 4: 88–95. 10.2174/1874613601004010088 20648225 PMC2905779

[44] Auerbach JD, Parkhurst JO, Caceres CF (2011) Addressing social drivers of HIV/AIDS for the long-term response: conceptual and methodological considerations. Glob Public Health 6 Suppl 3: S293–309. 10.1080/17441692.2011.594451 21745027

[45] Wechsberg WM, Luseno WK, Kline TL, Browne FA, Zule WA (2010) Preliminary findings of an adapted evidence-based woman-focused HIV intervention on condom use and negotiation among at-risk women in Pretoria, South Africa. J Prev Interv Community 38: 132–146. 10.1080/10852351003640799 20391060 PMC3129984

[46] Langen TT (2005) Gender power imbalance on women's capacity to negotiate self-protection against HIV/AIDS in Botswana and South Africa. Afr Health Sci 5: 188–197. 16245988 10.5555/afhs.2005.5.3.188 PMC1831928

[47] Jewkes R, Nduna M, Levin J, Jama N, Dunkle K, Puren A, et al (2008) Impact of stepping stones on incidence of HIV and HSV-2 and sexual behaviour in rural South Africa: cluster randomised controlled trial. BMJ 337: a506 10.1136/bmj.a506 18687720 PMC2505093

[48] Jewkes RK, Dunkle K, Nduna M, Shai N (2010) Intimate partner violence, relationship power inequity, and incidence of HIV infection in young women in South Africa: a cohort study. Lancet 376: 41–48. 10.1016/S0140-6736(10)60548-X 20557928

[49] Mugoya GC, Ernst K (2013) Gender differences in HIV-related stigma in Kenya. AIDS Care. 10.1080/09540121.2013.808733 23795954

[50] Hallman K (2004) Socioeconomic disadvantage and unsafe sexual behaviours among young women and men in South Africa Population Council.

[51] Maharaj P, Cleland J (2004) Condom Use Within Marital and Cohabiting Partnerships in KwaZulu‐Natal, South Africa. Studies in Family Planning 35: 116–124. 15260213 10.1111/j.1728-4465.2004.00013.x

[52] Johnson LF (2012) Access to Antiretroviral treatment in South Africa, 2004–2011. Southern African Journal of HIV Medicine: 22–27. 10.4102/sajhivmed.v18i1.694 PMC5843157 29568630

[53] Nattrass N (2008) Gender and access to antiretroviral treatment in South Africa. Feminist Economics 14: 19–36.

[54] Pool R, Montgomery CM, Morar NS, Mweemba O, Ssali A, Gafos M, et al (2010) Assessing the accuracy of adherence and sexual behaviour data in the MDP301 vaginal microbicides trial using a mixed methods and triangulation model. PLoS One 5: e11632 10.1371/journal.pone.0011632 20657774 PMC2908125

[55] Younge SN, Salazar LF, Crosby RF, DiClemente RJ, Wingood GM, Rose E, et al (2008) Condom use at last sex as a proxy for other measures of condom use: is it good enough? Adolescence 43: 927–931. 19149154 PMC2701210

[56] Noar SM, Cole C, Carlyle K (2006) Condom use measurement in 56 studies of sexual risk behavior: review and recommendations. Arch Sex Behav 35: 327–345. 16799837 10.1007/s10508-006-9028-4

[57] Gilbert C, Crook A, Nunn A, McCormack S (2008) Is self-reported sexual behaviour at the last sex act a good indicator of behaviour at all sex acts? [poster TB-401] Proceedings of Microbicides Conference. New Delhi, India.

[58] Gafos M, Ndlovu H, Mzimela M, team tM. How many women really achieve consistent condom use over the course of a year? Evidence from rural KwaZulu-Natal; 2010. pp. 22–24.

