# Correction: The Impact of Gender Norms on Condom Use among HIV-Positive Adults in KwaZulu-Natal, South Africa

**DOI:** 10.1371/journal.pone.0129637

**Published:** 2015-06-04

**Authors:** Kristin Fladseth, Mitzy Gafos, Marie Louise Newell, Nuala McGrath

S1 Appendix is inadvertently missing from the Supporting Information. It can be viewed below.

## Supporting Information

S1 Appendix. Appendix A.

## References

[1] Fladseth K, Gafos M, Newell ML, McGrath N (2015) The Impact of Gender Norms on Condom Use among HIV-Positive Adults in KwaZulu-Natal, South Africa. PLoS ONE 10(4): e0122671 doi: 10.1371/journal.pone.0122671 25853870 10.1371/journal.pone.0122671 PMC4390283

